# A 1-Day Training Course to Disseminate the BREF Psychoeducational Program to Caregivers and Promote Network Establishment between Psychiatry Departments and Family Associations

**DOI:** 10.1007/s40596-022-01632-1

**Published:** 2022-04-26

**Authors:** Romain Rey, Bénédicte de Martène, Matias Baltazar, Marie-Maude Geoffray, Thierry d’Amato, Caroline Demily, Anne-Lise Bohec

**Affiliations:** 1 University Claude Bernard Lyon 1, Lyon, France; 2 Le Vinatier Hospital, Bron, France

**Keywords:** Psychoeducation, Caregiver, Peer role-play, Education, Psychiatry

## Abstract

**Objective:**

Although international guidelines state that psychoeducation to caregivers should be provided systematically, it remains insufficiently available in psychiatry. This study reports the development and evaluation of an original training course aimed to provide participants with the knowledge and skills to implement “BREF,” a psychoeducational program for caregivers.

**Methods:**

The BREF program training course, a free, 1-day course incorporating peer role-play was developed. In addition to psychiatrists, nurses, and psychologists, caregivers were involved as preceptors. Participants were mental health professionals and volunteer caregivers. Participants to the first 28 sessions of the course (*n*=467) completed a post-course questionnaire (*n*=341) and a cross-sectional questionnaire (*n*=56). Quantitative data on satisfaction, learning, and behavior changes following the course were collected equating to levels 1, 2, and 3 of Kirkpatrick’s model.

**Results:**

After the course, high levels of satisfaction and commitment were observed with 100% of responders recommending the course and 81% intending to implement the BREF program. Confidence mean score to implement BREF was 7.9/10 (±1.4) with no significant effect of course session. At cross-sectional evaluation, 73% of responders reported improvements in skills related to providing psychoeducation to caregivers, 64% stated that the BREF program was implemented/under implementation, and 66% stated that their department had connected with a family association.

**Conclusions:**

Training course sessions alone can increase psychoeducational programs for caregivers and network establishment. The BREF program training course demonstrates a high level of participant satisfaction and is a promising method to disseminate psychoeducation to caregivers, thus addressing a major shortage in mental health organization.

Family caregivers fulfill multiple roles in the care of individuals with a severe mental disorder (SMD) such as schizophrenia, bipolar disorder, or major depressive disorder. In this regard, family caregivers are indispensable actors promoting recovery for individuals with a SMD [1]. While caring for someone with a SMD can be a positive experience, it is often associated with deleterious consequences on mental and physical health [2, 3]. Moreover, it is well established that both caregivers’ burden and caregivers’ coping abilities have an impact on patients’ recovery [4, 5]; it is thus of paramount importance to provide interventions to caregivers.

Psychoeducation to caregivers (PEC) aims at increasing caregivers’ knowledge and understanding of their relative’s illness and treatment. PEC also provides caregivers with skills to best support their relative and coping strategies to maintain their own well-being. PEC is associated with substantial beneficial outcomes on both individuals living with a SMD and their caregivers [6, 7]. It is thus considered an evidenced-based practice. Reviews and treatment guidelines therefore advocate that it should be provided to caregivers systematically and as early as possible after the onset of their relative’s disorder [8, 9].

Despite international and national guidelines advocating PEC as one of the most effective treatments for SMD, such programs remain scarce [10]. Only 3% of the 4.5 million French caregivers in psychiatry have benefited from PEC and less than 10% know family associations (i.e., peer-led organizations supporting family members, caregivers, and loved ones of individuals living with a SMD) [9]. Worryingly, PEC is provided on average 10 years after the disease onset in France. Recognizing this major shortage in mental health organization, Rey et al. created a short psychoeducational program called “BREF” [11]. BREF means “brief” in French and it can be provided early and systematically to caregivers of individuals with a SMD. Moreover, BREF encourages network establishment between psychiatry departments and family associations.

The present paper describes the development and evaluation of the BREF Program Training Course (BPTC) aimed to provide mental health professionals and volunteer caregivers of family associations with the knowledge and skills required to implement the BREF program in their psychiatry department.

## Methods

### Development of the BREF Program Training Course

#### Goals of the BPTC

The BPTC was designed with the aim to provide course participants with the knowledge and skills required to implement the BREF program in their psychiatry department. Additionally, the course aimed to (i) raise participants’ awareness regarding caregivers’ experience and needs, (ii) update participants’ knowledge on the efficiency of PEC in psychiatry, and (iii) encourage partnerships between psychiatry departments and family associations. On a broader level, the course was intended to help disseminate the BREF program nationally, therefore addressing shortages within the French mental health system. To our knowledge, no such training is currently available in France.

#### Continuing Education

Mental health professionals lack basic knowledge regarding interventions to caregivers [12, 13]. Including more courses about PEC in the initial training of mental health professionals might be seen as an easy way to promote early and systematic PEC. However, initial education is currently full. Moreover, we believe that the optimal time for mental health professionals to develop knowledge and skills related to PEC is when they are caring for individuals with a SMD professionally and have regular contacts with their caregivers. This could be achieved through continuing education.

#### Course Participants

Two distinct groups are eligible as course participants: direct providers of the BREF program and other key actors promoting recovery for individuals with a SMD.

As direct providers of the BREF program, nurses, psychologists, and volunteer caregivers of family associations are the natural audience of the BPTC. Including caregivers as course participants is critical since the BREF program is provided by mental health professionals together with volunteers of family associations. Indeed, the involvement of a peer co-leader is a key factor to improve effectiveness of PEC [14]. Moreover, volunteers of family associations are experienced caregivers with efficient coping skills, trained to provide peer support. Consistently with the recent French Council CESE instructions [9], we believe that training courses bringing together mental health professionals and volunteers of family associations have the ability to promote and facilitate network establishment.

Additionally, other key actors are eligible as course participants such as medical residents, senior psychiatrists, chief nurses, social workers, social educators, and medical secretaries. Although they do not provide the BREF program themselves, they are an appropriate audience for the BPTC because, as prescribers, managers, and/or essential actors supporting individuals with a SMD, they need to update their knowledge about PEC efficiency. Moreover, providing them with a clear understanding of the BREF program helps to implement it in their psychiatry department, thus promoting early and systematic PEC prescription.

#### Course Preceptors

All course directors (a psychiatrist, a psychologist, a nurse, and a volunteer caregiver from a family association) were experts in providing PEC and trained in family therapy. In addition to the course directors, a core group of course preceptors has been formed. This multidisciplinary group is composed of psychiatrists, nurses, and psychologists. Volunteer caregivers were also included in the group of course preceptors to take advantage of their experience, knowledge, and skills.

Consistently with the literature on consumer as teacher [15, 16], involving caregivers as teachers recognizes them as experts who should actively participate in the education of mental health professionals [17]. Caregivers as teachers help participants understand the feelings and emotions caregivers are dealing with when caring for their relative [18, 19]. They also provide knowledge about family associations. We thus believe that involving caregivers will promote positive changes in the mental health system [17].

To be included in the group of course preceptors, three requirements had to be fulfilled: (i) being a former participant of the BPTC, (ii) having provided the BREF program for 1 year at least, and (iii) co-providing the BPTC with an experienced preceptor. Although being trained to family therapy was valued, it was not compulsory to become a course preceptor since such professionals remain rare in the French public mental health system. Two to three preceptors were required to teach a BPTC session. It was essential to have enough course preceptors since we aimed to teach the BPTC frequently in order to disseminate the BREF program nationally.

#### Course Content and Format

All the course directors were involved in the design of the BPTC. The course content was conceived in accordance with recommendations on medical education curriculum regarding caregivers in psychiatry [13, 18]. The BPTC lasts 7 h and has been organized into 7 interventions during which exchanges are encouraged. After each intervention, 10 min is allotted to a discussion between course participants and preceptors. The course interventions, educational objectives, and teaching techniques are detailed in Table 1. Here, we develop the educational choices made to overcome the main hindrances to the development of PEC in France.

**Table 1:** Interventions topics, educational objectives, and teaching techniques of the BREF program training course

Intervention topics	Educational objectives	Teaching techniques
Caregivers in psychiatry PEC efficiency	- Explain the essential roles of caregivers in psychiatry - Describe the burden of caregivers supporting subjects with a SMD - Discuss the therapeutic benefits of PEC in psychiatry	Latest international guidelines
Caregivers’ experience and needs Family associations	- Identify the emotions and feelings experienced by caregivers in psychiatry - Recognize the impact of caregivers’ distress on the relative they are caring for - Identify caregivers’ expectations and needs - List the resources provided by family associations	Caregiver as a teacher Testimony of a caregiver Debate and exchanges
BREF program organization	- Explain the objectives and indications of the program - Justify the organization principles of the program - Describe the workflow of the program - Comply with the medical or professional secrecy issues	Lecture with slides Clinical illustrations Debate and exchanges
BREF program content and course	- Deliver the 3 sessions of the BREF program - Use the tools of the BREF program - Provide information and care to caregivers attending the BREF program - Deal with common issues encountered when providing PEC	Peer role-play Discussion in small groups Whole group reflection and exchanges Clinical illustrations, debates, brainstorming
Caregivers’ recovery process	- Explain caregivers’ need for various intervention levels over time - Identify the support and resources provided by health and social structures - Map and classify the various interventions to caregivers available in a health region - Create a local network through partnerships establishment with family associations	The Pyramid of Family Care template Discussion in small groups Whole group reflection and exchanges
Evaluation of PEC efficiency Participation to research projects	- Explain the purpose of the caregivers’ assessments incorporated in the BREF program - Interpret the scales and questionnaires of the BREF program - Use the data collection tools - Participate to prospective data collection on caregivers attending the BREF program	Efficiency of the BREF program
BREF network description	- Define the BREF network organization - Identify the support resources and devices provided by the BREF network	Tutorials about the digital resources

*PEC* psychoeducation to caregivers, *SMD* severe mental disorder

##### Addressing Providers’ Misconceptions

Common misconceptions explain mental health professionals’ reluctance to provide PEC. Indeed, PEC is wrongly seen as a complex intervention, requiring a high-level expertise and therefore not falling within mental health professionals’ competence. A peer role-play module was incorporated within the BPTC to overcome such misconstructions. Our intention was to have participants rehearse usual situations to improve their coping abilities before providing PEC [20]. Course participants were able to train in a safe setting and with the opportunity for a structured feedback. Moreover, playing the role of intervention recipients could help participants develop important communication skills, such as empathy [21].

##### Addressing Ethical and Privacy Concerns

Many mental health professionals fear that providing PEC could lead to a breach in professional secrecy. To overcome this common misconception, a substantial amount of time throughout the BPTC was allotted to exchanges and debates between participants and preceptors. Questions about medical secrecy were systematically encouraged and addressed. We hoped that such a content and interactive format would help participants feel able to provide PEC and convince them that early and systematic provision of PEC is an essential duty for any mental health professional.

##### Addressing Providers’ Poor Awareness Regarding Caregivers

Not only mental health professionals have little knowledge about caregivers’ needs and experience, but they are not aware of the resources and support interventions provided by family associations. Two choices were made to address these issues. Firstly, we believed that involving caregivers *as course preceptors* would be engaging for the course participants and would encourage them to provide PEC, establish partnerships with, and refer caregivers to family associations. Secondly, we hoped that including caregivers *as course participants* would promote a sense of partnership between caregivers and mental health professionals. Engaging caregivers within the education process and care provision could improve the referral to family associations, thereby increasing the number of caregivers receiving peer support.

##### Addressing Referral Issues

Adequately referring caregivers after the BREF program is of crucial importance since some interventions such as systemic, cognitive-behavioral, or medical family therapies although highly efficient remain rare in France. To this end, we designed an interactive exercise based on the Pyramid of Family Care [22]. This model encourages course participants to (i) identify the different resources for caregivers locally available, (ii) classify them into 5 levels corresponding to situations of increasing complexity, and (iii) connect with the associations and structures providing them. Eventually, this exercise provides course participants with a user-friendly template to refer caregivers to the most appropriate intervention.

##### Addressing the Low Priority Given to PEC Trainings

The lack of knowledge about PEC efficiency and the limited funding explain the low priority currently given to PEC trainings in France. In this challenging context, we believed that a free, 1-day format would improve the admissibility of the BPTC, allow us to teach it frequently, and, ultimately, help disseminate the BREF program nationally.

### Evaluation of the BREF Program Training Course

#### Objectives

The BPTC was assessed in accordance with Kirkpatrick’s 4-level training evaluation model, one of the most widely used frameworks to evaluate health education programs [23]. Under this model, training can be assessed across 4 different levels, i.e., reaction, learning, behavior, and results. In this paper, we report results equating to levels 1, 2, and 3 of Kirkpatrick’s model. First, we assessed participants’ satisfaction with the learning experience (level 1, reaction). Then, we evaluated their confidence, commitment, and perceived skills improvement following the course (level 2, learning). Furthermore, since the BPTC was taught frequently and by various course preceptors, we looked for variability in participants’ confidence to implement the BREF program across all the sessions of the BPTC. Lastly, we explored participants’ behavioral changes transferred to their psychiatry service (level 3, behavior).

#### Course Assessments

Following the BPTC, course participants were asked to complete two questionnaires. Participants’ informed consent was sought prior to data collection, and information was provided on researchers’ contact details, anonymity, and right to withdraw. Hence, in accordance with French regulations on educational research, Le Vinatier Hospital Center Institutional Review Board (IRB) Office determined the project was exempt from IRB review.

The post-training course questionnaire, an anonymous, self-administered questionnaire, was completed by the participants immediately after the course. Five questions assessed participants’ satisfaction level using a 3-point Likert scale with 1 being “satisfied,” 2 being “neutral,” and 3 being “not satisfied.” Two additional questions explored participants’ confidence and commitment.

The cross-sectional questionnaire was sent on the 5th of January 2021 to participants who provided their mail contact. Using Framaforms [24], this anonymous web questionnaire invited participants to self-assess the BPTC impact on their skills and behaviors. One question explored changes in skills related to PEC provision. Behavioral changes were assessed through three questions relating to (i) progress of the BREF program implementation and (ii) connection with local family associations.

#### Statistical Analysis

JASP software (JASP Team (2019), JASP (Version 0.14) [Computer software]) was used to perform statistical analyses. Categorical variables are presented as the number of responders and percentages (*n*; %). Quantitative variables are presented as the mean and standard deviation (m ± SD). Proportion comparisons were performed using the chi-square test. Significance threshold for the statistical tests was fixed at *p*<0.05.

Analyses regarding the effect of session on the confidence to implement the BREF program were performed using the R software [25]. We computed a Linear Mixed Model using the lmer command of the lme4 package [26] to assess the impact of session on the confidence to implement the BREF program (our primary variable of interest). Session was entered as a categorical random effect. The 1 to 10 ordinal scale was treated as a numerical variable. The alpha value was set at 0.05. Data were plotted using ggplot2 [27].

## Results

Between October 2018 and September 2020, 28 sessions of the BPTC were realized in France, totalizing 467 participants. Here, we report the results obtained from the two questionnaires administered to participants of the 1st to 28th sessions of the BPTC.

### Descriptive Characteristics of the Responders

The post-training course questionnaire was returned by 403 (86.3%) of the 467 participants who underwent the BPTC. After discarding 61 unusable questionnaires (blank questionnaires), 341 responders were included in this study. The 341 responders were mostly women (81%) and belonged to 56 different employing-organizations, 90% were mental health professionals, and the remaining 10% were volunteer caregivers of family associations. The cross-sectional questionnaire was completed by 56 (16.4%) of the 341 participants who completed the post-training course questionnaire. Responders were mostly women (73%) and belonged to 56 different employing-organizations, 89% were mental health professionals, and the remaining 11% were volunteer caregivers of family associations. There was no difference in gender (*χ*^2^=1.940, *df*=1; *p*=0.164), professional activity (*χ*^2^=0.029, *df*=1; *p*=0.864), and type of employing-organization (*χ*^2^=3.313, *df*=4; *p*=0.507) between the two groups of responders.

### Main Findings on Participants’ Satisfaction (Kirkpatrick’s Level 1)

Analysis of the post-training course questionnaires showed that 100% of responders would recommend the training course to others. Responders also reported high levels of satisfaction regarding the material organization, teaching techniques, knowledge provided, and preceptors’ lectures (93%, 94%, 70%, and 98% respectively).

### Main Findings on Participants’ Learning (Kirkpatrick’s Level 2)

Analysis of the post-training course questionnaires showed high levels of confidence and commitment. Following the BPTC, responders’ confidence to implement the BREF program was high with a mean score of 7.9/10 (±1.4). Consistently, 81% of responders intended to implement the BREF program in their psychiatry department. Remarkably, the effect of course session on the confidence to implement the BREF program was not significant (*χ*^2^_16_=18.9; *p*=0.27). Analysis of the cross-sectional questionnaires showed that 73% of responders reported an improvement in their ability to provide PEC following the training course. Moreover, responders deemed that the BPTC had a very positive impact on their professional practices with a mean score of 7.9/10 (± 1.7).

### Main Findings on Participants’ Behaviors (Kirkpatrick’s Level 3)

At cross-sectional evaluation, 64% of responders stated that the BREF program was implemented or under implementation in their psychiatry department. Responders deemed the BREF program implementation as moderately easy with a mean score of 6.2/10 (±2.3). Regarding network establishment, 66% of responders reported that their psychiatry department had connected with a family association following the training course.

## Discussion

The results of the present study have five important implications regarding the development of educational initiatives around PEC.

Firstly, the course participants reported a high level of satisfaction regarding the various characteristics of the training course. Notably, 100% of responders would recommend the course to other mental health professionals. To date, we are unaware of any research of similar size assessing participant satisfaction of a training course about PEC. Furthermore, 98% of responders deemed to be satisfied with the preceptors’ lectures, suggesting that no resistance arose from the involvement of caregivers as course preceptors. Such a high satisfaction level strengthens the interest of engaging caregivers within medical education initiatives [17].

Secondly, our findings support the value of the BPTC for improving participants’ skills, confidence, and commitment in providing PEC in the short and medium terms. Immediately after the training course, very satisfactory data regarding responders’ confidence and commitment to implement the BREF program were observed. At cross-sectional evaluation as well, the majority of responders reported an improvement in skills related to PEC provision. This is in accordance with the efficacy of a short interprofessional training course in improving clinicians’ confidence and abilities in working with families of individuals with a SMD [28].

Thirdly, the present study indicates that the BPTC helps mental health professionals overcome their reluctance in working with caregivers. Indeed, despite the clear benefits in providing interventions to caregivers [1, 29], resistance from mental health professionals persists [17]. Encouragingly, responders deemed that the BPTC had a very positive impact on their professional practices (mean score ± SD: 7.9/10 ± 1.7). Moreover, 81% of responders intended to implement the BREF program in their psychiatry department after the course, suggesting that the BPTC helped to dispel professionals’ common erroneous believes that caregivers’ involvement can be harmful, unnecessary, and laborious [30–32].

Fourthly, the BPTC was enough to implement the BREF program which was the main goal of the course. Immediately following the course, responders reported a high level of confidence to implement the BREF program in their psychiatry department (mean score ± SD: 7.9/10 ± 1.4). Furthermore, although some participants attended the BPTC only 3 months before the cross-sectional evaluation, 64% of the cross-sectional questionnaire responders stated that the BREF program was implemented or under implementation and 66% that their psychiatry department had connected with a local family association. Responders deemed the BREF program implementation as moderately easy with a mean score of 6.2/10 (±2.3). Further evaluation is ongoing to identify the main barriers of the BREF program implementation.

Our data confirm that a 1-day format is enough to implement the BREF program. To date, no study evaluating a short training course aimed to develop PEC knowledge and skills is available. However, our results are consistent with previous reports that 1-day courses are effective in improving knowledge, skills, and confidence regarding key non-pharmacological treatments in gerontology [33] or psychiatry settings [28].

To address providers’ misconceptions regarding PEC, we incorporated a peer role-play module (a simulation-based training method) within the BPTC. Although our evaluation data did not assess the specific effect of peer role-play in the global impact of the BPTC, our results raised the question whether it is a key factor in the effectiveness of our training course. Indeed, simulation-based training is now considered a core educational strategy in mental health [34, 35]. It is not only an effective method to develop knowledge and skills for working with families in psychiatry, but also an efficient way to help clinicians develop new ways of working [28]. Furthermore, simulation-based training can develop practical educational aspects that are difficult to adequately address during lectures [36]. Future evaluations of the BPTC are needed to address the specific impact of the peer role-play module.

Fifthly, the BPTC alone led to an increase in the structures providing the BREF program and to network establishment in France. Indeed, at cross-sectional evaluation, 64% of responders stated that the BREF program was implemented/under implementation and 66% that their department had connected with a family association. This is consistent with the previous report that a 1-day training workshop is an effective method to disseminate cognitive stimulation therapy (another non-pharmacological treatment) in dementia services [33]. Furthermore, we showed that the BPTC can be taught by various course preceptors without significant variability in the participant’s confidence to implement the BREF program following the training. This is especially encouraging since the broader goal of the BPTC was to disseminate the BREF program nationally, therefore addressing shortages within the French mental health system.

Although created in France, the present model could prove relevant in other countries. Indeed, both the BREF program and the BPTC were conceived in accordance with international recommendations [13, 18, 22]. Furthermore, they aim at addressing the initial and priority needs of caregivers in psychiatry which are non-specific to France [22]. Finally, 1-day training courses are effective methods to disseminate non-pharmacological interventions and new ways of working in various countries [28, 33]. Subject to the taking into account of national peculiarities (such as variations in health system organization and family association networks) or cultural differences, we therefore believe that the present model could generalize outside of France. Attempts to disseminate the BREF program using the BPTC are currently scheduled for Belgium and under consideration for Morocco.

In interpreting the findings of this study, a number of limits need to be considered. Firstly, some advanced skills related to PEC provision could not be included in the course content. Indeed, although communication skills, problem solving approaches, or cognitive-behavioral techniques are key elements in psychoeducational programs, teaching them to the BPTC participants was not compatible with a 1-day course format. To compensate for this missing content, complementary e-learning modules are in preparation and will be made available to all former course participants.

Secondly, only post-course questionnaires were used to assess the BPTC. To better identify specific achievements and shortcomings of the course, a comparison of performance levels of individual students before and after course participation is needed. Furthermore, prior knowledge and skills were not explored. Such an assessment allows a more accurate evaluation of learning improvements after the BPTC since participants may have differing performance levels when entering the course. Finally, while we explored satisfaction, skills, and behavior improvement, we did not evaluate changes in knowledge following the BPTC. Given these limits, since April 2021, new evaluations are ongoing to further assess the BPTC. We designed new pre- and post-course questionnaires exploring knowledge and skills. Additionally, the pre-course questionnaire allows participants to self-assess their prior knowledge and skills.

Thirdly, we used self-administered questionnaires which are widely used in health training research [37], although their accuracy has been questioned as compared to objective measures. Nevertheless, a good agreement between self-administered evaluation and objective measures of performance has been recently reported, suggesting that self-administered questionnaires are a valid tool to assess student performance on specific learning objectives [38].

Fourthly, the quantitative measures used in the post-course and cross-sectional questionnaires were specifically developed for the evaluation of the course; they were thus not validated. In the absence of existing validated instruments to assess the BPTC [39], the course directors developed questionnaires with consideration of guidelines regarding health education evaluation [37, 39]. To improve content validity, the questions were designed to explore the main educational objectives of the BPTC. Moreover, to strengthen the measures’ validity, questions evaluating participants’ behavioral changes explored tangible outcomes such as implementation of the BREF program or connection with local associations.

Lastly, 61 of the 403 returned post-training course questionnaires were discarded because they were unusable (blank questionnaires) and only a subset of the course participants completed the cross-sectional web questionnaire. Our results can thus be subjected to sampling bias since low-performing students are less likely to participate in online evaluations as compared to their high-performing peers [40]. However, there was no significant difference in gender, professional activity, and type of employing-organization between post-training course and cross-sectional questionnaire responders.

In conclusion, in addition to showing a high level of participant satisfaction, our 1-day training course helps overcome resistance in working with caregivers and is sufficient to implement the BREF program. BPTC sessions alone can lead to an increase in psychoeducational programs for caregivers and network establishment between psychiatry departments and family associations. In this regard, the BPTC is a promising method to disseminate PEC, thus addressing a major shortage in mental health organization.
